# Baricitinib treatment for hospitalized patients with severe COVID-19 on invasive mechanical ventilation: a propensity score-matched and retrospective analysis

**DOI:** 10.3389/fmed.2025.1445809

**Published:** 2025-01-22

**Authors:** Yanxiong Mao, Anyi Guo, Ying Zhang, Jianxing Lai, Dian Yuan, Hao Zhang, Wenqi Diao, Weisong Chen, Fugui Yan

**Affiliations:** ^1^Key Laboratory of Respiratory Disease of Zhejiang Province, Department of Respiratory and Critical Care Medicine, Second Affiliated Hospital of Zhejiang University School of Medicine, Hangzhou, Zhejiang, China; ^2^Department of Scientific Research, Second Affiliated Hospital of Zhejiang University School of Medicine, Hangzhou, Zhejiang, China; ^3^Department of Respiratory, Jinhua Municipal Central Hospital, Jinhua, Zhejiang, China

**Keywords:** COVID-19, baricitinib, mortality, propensity score matching, invasive mechanical ventilation

## Abstract

**Introduction:**

Baricitinib is a selective inhibitor of Janus kinase (JAK)1 and JAK2, which is associated with clinical improvement in non-severe COVID-19 patients. But in severe COVID-19 patients, the effectiveness of baricitinib is still controversial.

**Methods:**

A propensity score-matched and retrospective study was conducted to evaluate the effectiveness of baricitinib in severe COVID-19 patients requiring invasive mechanical ventilation (IMV).

**Results:**

A total number of 48 patients treated with baricitinib were included, and 48 patients were assigned to control group by propensity score matching. The mean ages were high in both group (baricitinib group vs. control group: 78.80 ± 9.04 vs. 82.57 ± 9.27), and most were unvaccinated (62.5% vs. 66.7%. Baricitinib group had a higher proportion of patients with hypertension (73.9% vs. 45.5%, *p* = 0.006). Control group had higher level of creatine kinase-myocardial band (247.50 vs. 104.50, *p* = 0.021). Patients in the baricitinib group were more likely to receive nirmatrelvir/ritonavir (39.6% vs. 16.7%, *p* = 0.017) and intravenous immunoglobin (14.6% vs. 0, *p* = 0.007). Baricitinib group had significantly lower all-cause 28-days mortality than control group (72.9% vs. 89.6%, *p* = 0.004).

**Conclusion:**

The present study revealed baricitinib reduced 28-days mortality in severe COVID-19 patients on IMV. The effectiveness of baricitinib in treating patients with severe COVID-19 on IMV needs to be further investigated through future studies.

## Introduction

Since 2019, the COVID-19 pandemic has represented a major cause of mortality worldwide. People of all ages are susceptible to COVID-19 (1). However, elderly patients are more likely to develop severe COVID-19 infection that requires invasive mechanical ventilation (IMV) (2, 3). There are various therapeutic options for COVID-19 including antiviral drugs, glucocorticoids, cytokine antagonists, Janus kinase (JAK) inhibitors and so on (4). Antiviral drugs target viral proteins to block the virus life cycle, but most of them need the administration in early stage of symptom onset (4, 5). Glucocorticoids, which are known to reduce the mortality among severe COVID-19 patients, are recommended mostly for inpatients with COVID-19 who are receiving oxygen support (6). Tocilizumab, an interleukin (IL)-6 receptor antagonist, in combination with glucocorticoids, reduces mortality in patients with severe or critical COVID-19 (7). Before COVID-19 pandemic, JAK inhibitors were used alone or with other medications to treat rheumatoid arthritis (8). The use of JAK inhibitors in COVID-19 were tested by various studies, and baricitinib stood out for its effectiveness and received attention from researchers and physicians (7, 9).

Baricitinib was a selective inhibitor of JAK1 and JAK2. In early 2020, artificial intelligence identified baricitinib as a potential intervention for COVID-19 due to its known anti-cytokine properties and potential for targeting host proteins for its antiviral mechanism (10, 11). Following the identification, numerous studies investigated the potential use of baricitinib in COVID-19 and provided substantial evidence of clinical improvement associated with baricitinib in non-severely hospitalized patients (12–14). The phase 3 COV-BARRIER study found that baricitinib, in addition to standard of care, was associated with reduced mortality in hospitalized patients with COVID-19, and had a similar safety profile to that of standard of care alone (10). The RECOVERY study revealed that baricitinib significantly reduced the risk of death in patients hospitalized with COVID-19 (15). With established safety profiles, baricitinib offers potential benefits for inpatients with COVID-19, especially the elderly (4). Based on substantial evidence, the use of baricitinib was recommend in hospitalized patients with COVID-19 (13).

But in severe hospitalized patients with COVID-19, the effectiveness of baricitinib was still controversial. On one hand, some studies found that baricitinib reduced mortality in severely ill patients. A small sample size exploratory trial showed that baricitinib reduced mortality in critically ill hospitalized patients with COVID-19 on IMV or extracorporeal membrane oxygenation (ECMO) (16). On the other hand, some studies showed that baricitinib brought no benefit on the mortality in severely ill patients. In the Bari-SolidAct study, no statistically significant difference was observed on 60-day mortality in hospitalized patients with severe/critical COVID-19 receiving either baricitinib or placebo (17). In subgroup analysis of RECOVERY study, baricitinib didn’t reduce mortality among patients on IMV (15). So the effect of baricitinib on severe COVID-19 was still controversial, more research was needed to draw a definite conclusion. To answer the question, further phase 3 randomized controlled trials (RCT) with large sample size are warranted. However along with massive vaccination across the globe, the incidence of severe COVID-19 is in decline, which makes the phase 3 RCT with large sample size less feasible. So the real-world studies, which have a mutually supplementary relationship with RCT, might be a good alternative. Real-world studies, which reflects the actual clinical aspects, could use data from collected from diversified areas including Electric Medical Record System (EMRS) used in hospitals. EMRS data, which are recognized as definitive data with the highest reliability among real-world data, are increasingly used for medical research (18). It is reasonable to assume that retrospective real-world studies by using EMRS data may provide further evidence of use of baricitinib in severe hospitalized patients with COVID-19. So we aimed to evaluate the effectiveness of baricitinib in severe COVID-19 patients requiring IMV in a propensity score-matched and retrospective study.

## Methods

### Study population

All patients admitted to the study hospital between December 1st 2022 to January 31st 2023 with discharge diagnosis of COVID-19 infection were retrieved from EMRS. During this time period, China had a policy shift in COVID-19 control, and our tiered medical system had dealt with a rise in infections in the country.

Two pulmonologists (AYG and HZ) reviewed the cases one by one independently and identified severe COVID-19 patients on IMV. Where differences arose, the third pulmonologist (JXL) validated the assessment. The standard of the reviewing was based on the following inclusion criteria and exclusion criteria. The inclusion criteria were as follows: (1) aged 18 years or older; (2) laboratory-confirmed COVID-19 infection; (3) use of IMV; (4) evidence of pneumonia or clinical symptoms of COVID-19. The exclusion criteria were as follows: (1) unknown vaccination status of COVID-19; (2) hospital-acquired COVID-19 infections; (3) intubated prior to admission.

Treatment strategies were based on institutional protocols that were updated weekly by a multidisciplinary panel, based on national COVID-19 guidelines published by National Health Commission of the People’s Republic of China. All severe COVID-19 patients on IMV received standard of care therapies, which included corticosteroids and prone position ventilation. The use of antiviral drugs, baricitinib, tocilizumab, intravenous immunoglobin (IVIG) or ECMO were left at the discretion of the physicians. During the study period, the Omicron variant of COVID-19 was the dominant variant in our province.

### Data collection and outcomes

The primary outcome of current study was 28-days all-cause mortality after admission. Data were extracted from EMRS by a combination of drug utilization reports and manual chart reviews. Demographics, vaccination status, lab test results on admission, disease comorbidities, and pharmacotherapy were collected.

### Propensity score matching

Patients who were treated with baricitinib during their hospital stay were defined as patients of baricitinib group. Per hospital’s treatment strategy, baricitinib 4 mg daily was the standard dose. To ensure a valid comparison of similar patients, we then used propensity score matching to 1:1 match patients treated with baricitinib and control. The following covariates were used for the calculation of the propensity score: age, sex, vaccination status, heart failure, renal insufficiency, diabetes mellitus, rheumatoid disease, interstitial lung disease, chronic obstructive pulmonary disease (COPD) and history of malignancy.

### Statistical analysis

The results were analyzed using IBM SPSS Statistics 20. Continuous data was presented as the mean with stand deviation (SD) or median with interquartile range (IQR), depending on the distribution of data. For continuous data before PSM, if the data followed the normal distribution, variables were compared using Student’s *t*-test for independent samples. If the data didn’t follow the normal distribution, Mann-Whitney U test was used.

For continuous data after PSM, if the data followed the normal distribution, variables were compared using Student’s *t*-test for paired samples. If the data didn’t follow the normal distribution, Wilicoxon signed-rank for paired samples test was used. Categorical data were presented as absolute value and percentage. For categorical data before PSM, Chi-square test was used. For categorical data after PSM, McNemar test for paired data was used. Kaplan-Meier curves were used for survival analysis, and a dependent samples log-rank test was used to compare the curves. Statistical significance was set at *p* < 0.05.

## Results

**FIGURE 1:**
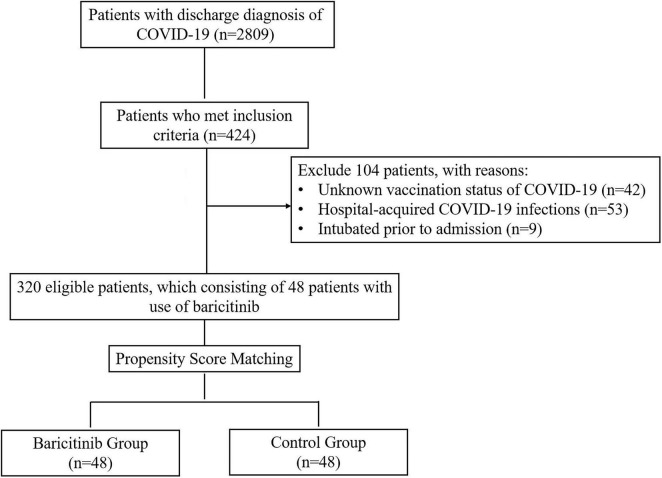
Flow chart of study population. COVID-19: coronavirus disease 2019.

Between December 1st 2022 to January 31st 2023, a total number of 2809 patients with discharge diagnosis of COVID-19 infection were identified from EMRS. After review, 424 patients met the inclusion criteria. Of those 424 patients, 104 patients were excluded. A final number of 320 patients were eligible for further analysis, which consisting of 48 patients treated with baricitinib. After PSM, 48 patients were assigned to control group (Figure 1). Covariates used for the calculation of the propensity score were compared before and after PSM (Table 1). The mean participant age were high in both group (baricitinib group vs. control group: 78.80 ± 9.04 vs. 82.57 ± 9.27). Most patients were unvaccinated in both groups (baricitinib group vs. control group: 62.5% vs. 66.7%). Both groups had a large proportion of patients with heart failure (baricitinib group vs. control group: 39.6% vs. 47.9%) and renal insufficiency (39.6% vs. 41.7%). Baseline demographic and comorbidity data were balanced between two groups, except for hypertension. Compared to control group, baricitinib group had a higher proportion of patients with hypertension (70.8% vs. 41.7%, *p* = 0.029) (Table 2).

**TABLE 1:** Baseline patient characteristics before and after propensity score match.

	Before propensity score match			After propensity score match		
	**Baricitinib group (*n* = 48)**	**Control group (*n* = 272)**	***p***	**Baricitinib group (*n* = 48)**	**Control group (*n* = 48)**	***p***
Age, years	78.28 (8.86)	79.20 (9.22)	0.77	78.80 (9.04)	82.57(9.27)	0.054
Male	29 (60.4%)	194 (71.3%)	0.05	27 (56.2%)	23 (47.9%)	0.54
COVID-19 vaccination status			0.647			0.716
Unvaccinated	30(62.5%)	152 (55.9%)		30 (62.5%)	32 (66.7%)	
1 shot	5 (10.4%)	26 (9.6%)		5 (10.4%)	3 (6.3%)	
2 shots	5 (10.4%)	32 (11.8%)		5 (10.4%)	4 (8.3%)	
3 shots	8 (16.7%)	62 (22.8%)		8 (16.7%)	9 (18.8%)	
Congestive heart failure	19 (39.6%)	91 (33.5%)	0.557	19 (39.6%)	23 (47.9%)	0.297
Renal insufficiency	19 (39.6%)	131 (48.2%)	0.171	19 (39.6%)	20 (41.7%)	0.691
Diabetes mellitus	9 (18.8%)	94 (34.6%)	0.019	9 (18.8%)	11 (22.9%)	0.535
Interstitial lung disease	2 (4.2%)	15 (5.5%)	0.652	2 (4.2%)	2 (4.2%)	0.964
Chronic obstructive pulmonary disease (COPD)	1 (2.1%)	28 (10.3%)	0.058	1 (2.1%)	2 (4.2%)	0.531
History of malignancy	6 (12.5%)	46(16.9%)	0.375	6 (12.5%)	4 (8.3%)	0.779

Values are expressed as mean value ± standard deviation or as number (percentage) of subjects.

**TABLE 2:** Comparison of baseline demographic and comorbidity data between baricitinib group and control group.

Variables	Baricitinib group (*n* = 48)	Control group (*n* = 48)	*p*
BMI, kg/m^2^	23.09 (3.78)	22.00 (3.73)	0.337
Smoking status			0.337
Non-smoker	32 (69.6%)	38 (86.4%)	
Ex-smoker	6 (13.0%)	5 (11.4%)	
Smoker	8 (17.4%)	1 (2.3%)	
Alcohol drinking status			0.539
Non-drinker	34 (73.9%)	39 (88.6%)	
Ex-drinker	6 (13.0%)	4 (9.1%)	
Drinker	6 (13.0%)	1 (2.3%)	
**Comorbidity**			
Asthma	0	0	–
Hepatic disease	3 (6.2%)	3 (6.2%)	–
Cerebrovascular disease	5 (10.4%)	5 (10.4%)	–
Hypertension	34 (70.8%)	20 (41.7%)	**0.029**

Data are mean (SD), median (IQR), median, or n (%). BMI, body mass index. Bold means *p* < 0.05.

The time from admission to intubation was similar between groups (Table 3). Regarding biomarkers of infection, both groups had similar levels of C-reactive protein (CRP), IL-6 and procalcitonin, which suggested that the severity of COVID-19 infection were similar between groups. Control group had higher level of creatine kinase-myocardial band (CK-MB) than baricitinib group (247.50 vs. 104.50, *p* = 0.021). But both groups had similar level of creatine kinase (CK). There was no significant difference in arterial pressure of oxygen and arterial pressure of carbon dioxide between two groups.

**TABLE 3:** Comparison of clinical expression between baricitinib group and control group.

Variables	Baricitinib group (*n* = 48)	Control group (*n* = 48)	*p*
Time from admission to intubation, days	1 (0, 4)	1 (0, 5)	0.506
**Infection biomarkers**			
CRP, mg/L	80.15 (29.13, 128.45)	94.15 (50.50, 134.45)	0.236
IL-6, pg/mL	48.47 (21.90, 152.20)	235.55 (56.60, 598.68)	0.069
Procalcitonin, ng/mL	0.24 (0.14, 0.86)	0.62 (0.14, 4.38)	0.179
**Blood cells count**			
White blood cell count, ×10^9^/L	11.17 (14.13)	9.09 (5.75)	0.454
Neutrophil count, ×10^9^/L	8.32 (4.92)	7.75 (5.42)	0.413
Lymphocyte count, ×10^9^/L	2.41 (12.07)	0.81 (0.65)	0.963
Hemoglobin, g/L	121.35 (20.91)	125.89 (24.09)	0.971
Platelets count, ×10^9^/L	155.20 (77.01)	166.57 (70.64)	0.903
Albumin, g/L	30.89 (4.91)	31.26 (5.11)	0.921
CK-MB, U/L	104.50 (53.00, 297.75)	247.50 (107.25, 505.75)	**0.021**
CK, U/L	21.50 (50.44)	24.57 (32.18)	0.693
D-dimer, μg/L	1865.00 (920.00, 2805.00)	2245.00 (1092.50,4140.00)	0.492
**Arterial blood gas analysis**			
PaO_2_, mmHg	84.60 (66.70, 103.0)	83.80 (70.30, 141.75)	0.330
PaCO_2_, mmHg	34.40 (28.85, 39.05)	31.65 (27.88, 36.65)	0.837

Data are mean (SD), median (IQR), median, or n (%). CRP, C-reactive protein; pro-BNP, pro-brain natriuretic peptide; PaO_2,_ partial pressure of oxygen; PaCO_2_, partial pressure of carbon dioxide; CK-MB, creatine kinase-myocardial band; CK, Creatine kinase. Bold means *p* < 0.05.

All patients in both groups received systemic corticosteroids, and the daily dose of systemic corticosteroids was similar between groups (Table 4). The majority in both groups received at least one of the three antiviral drugs including nirmatrelvir/ritonavir, azvudine and molnupiravir (baricitinib group vs. control group: 81.2% vs. 72.9%). Baricitinib group had a significant higher proportion of patients received nirmatrelvir/ritonavir than control group (39.6% vs. 16.7%, *p* = 0.017), and higher proportion of patients who switched antiviral drugs during treatment (31.2% vs. 12.5%, *p* = 0.033). There was no significant difference in use of antibiotics, low molecular weight heparin, tocilizumab and ECMO between the two groups. But patients in the baricitinib group were more likely to receive IVIG (14.6% vs. 0, *p* = 0.007).

**TABLE 4:** Comparison of treatment and outcome between baricitinib group and control group.

Variables	Baricitinib group (*n* = 48)	Control group (*n* = 48)	*p*
Antiviral treatment	39 (81.2%)	35 (72.9%)	0.516
Nirmatrelvir/ ritonavir	19 (39.6%)	8 (16.7%)	**0.017**
Azvudine	18 (37.5%)	13 (27.1%)	0.339
Molnupiravir	19 (39.6%)	20 (41.7%)	0.691
Antiviral drugs switched	15 (31.2%)	6 (12.5%)	**0.033**
Antibiotic use	48 (100%)	43 (89.6%)	0.304
LMWH use	41 (85.4%)	37 (77.1%)	0.482
Tocilizumab use	1 (2.1%)	2 (4.2%)	0.531
Immunoglobulin use	7 (14.6%)	0	0.007
ECMO use	1 (2.1%)	0	0.325
**Corticosteroids**			
Systemic corticosteroids use	48 (100%)	48(100%)	–
Daily dose of systemic corticosteroids, mg/day^#^	187.06 (135.00, 221.03)	168.33 (152.94, 200.00)	0.170

Data are mean (SD), median (IQR), median, or n (%). LMWH, Low molecular weight heparin.

^#^Reported in Methylprednisolone equivalent; ECMO, extracorporeal membrane oxygenation. Bold means *p* < 0.05.

**FIGURE 2:**
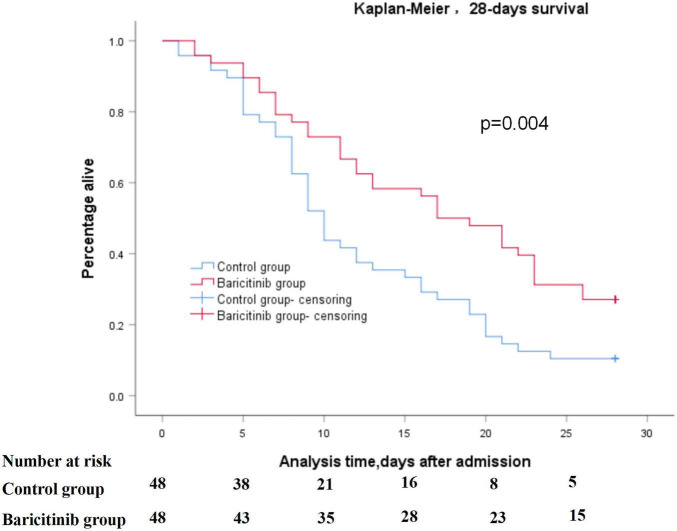
Kaplan-Meier analysis of survival to 28-days after admission between two groups.

Both groups reported high all-cause 28-days mortality (baricitinib group: 72.9% vs. control group: 89.6%). The Kaplan-Meier analysis identified significant difference between two groups in all-cause 28-days mortality after admission (log rank, *p* = 0.004) (Figure 2).

## Discussion

Our observational study compared real-world outcomes with baricitinib versus control for treatment in patients hospitalized with severe COVID-19 on IMV in a propensity score-matched and retrospective cohort. The study found high mortality among patients hospitalized with severe COVID-19 on IMV. The result suggested that baricitinib reduce 28-days mortality in severe COVID-19 patients on IMV.

One of the major strengths of our study was the PSM design. There are a few known risk factors for severe COVID-19, which could have impact on prognosis in a retrospective study. So far, age and comorbidities such as diabetes, COPD, chronic kidney disease, have been reported to be independent predictors of mortality for COVID-19 patients (3, 19–24). Vaccine has been shown to bring survival benefit in COVID-19, which can also influence the outcomes (25, 26). With the expanding coverage of COVID-19 vaccination, its influence on reducing mortality could not been overlooked. So the current study excluded patients with unknown vaccination status. Given these potentially confounding variables and the retrospective, observational nature of current study, PSM was applied to minimize bias. Therefore, the outcomes were assessed between two comparable groups, which increased validity of our study. Moreover in our study, the infection biomarkers such CRP, IL-6 and procalcitonin, were balanced between both groups, which suggested that the severity of infection was similar. So the balanced baseline demographics, comorbidity profile and severity of infection lend credibility to our findings.

Our study showed a significant difference in the 28-days all-cause mortality between severe COVID-19 patients on IMV treated with baricitinib versus control. In less severe hospitalized COVID-19 patients, the benefit of baricitinib on reducing mortality was reported by numerous studies. It was roughly estimated that baricitinib or another JAK inhibitor was associated with a 20% proportional reduction in mortality (15). However, in severe COVID-19, there were discrepant results about effectiveness of baricitinib among studies. On one hand, some studies found that baricitinib reduced mortality in severely ill patients. In an exploratory trial conducted in severe patients on IMV or ECMO, treatment with baricitinib compared with placebo reduced 28-days all-cause mortality by 46% and 60-day all-cause mortality by 44% (16). But this trial had a relatively small sample size. On the other hand, some studies showed that baricitinib brought no benefit on the mortality in severely ill patients. In the Bari-SolidAct study, no statistically significant difference was observed on 60-days mortality in hospitalized patients with severe/critical COVID-19 receiving either baricitinib or placebo (17). Of note, the trial was stopped before reaching planned sample size, so the results should be treated with caution. In subgroup analysis of RECOVERY study, baricitinib didn’t reduce mortality among patients on IMV (15). Our findings provided a new support of the use of baricitinib in severe COVID-19 patients on IMV. But the effectiveness of baricitinib in severe COVID-19 was still needed more research to draw a definite conclusion.

Despite the use of standard care, our study reported high mortality of 72.9% for baricitinib group and 89.6% for control group, which were much higher than reported in previous studies. In an exploratory trial conducted in severe patients, the overall 28-days all-cause mortality for those on IMV or ECMO at baseline was 39% in participants who received baricitinib and 58% in those who received placebo (16). The open-label RECOVERY study reported that 28-days mortality of 49% in participants who received tocilizumab while on IMV versus 51% in those who received standard of care (15). In one large meta-analysis, the case fatality rate of 45% was reported for patients with severe COVID-19 requiring IMV (16). This discrepancy in mortality may be explained by the following reasons. First, our study included older patients than previous studies. In our study, the mean age was 78.80 ± 9.04 years for baricitinib group and 82.57 ± 9.27 years for control group. The mean participant age was 58.6 ± 13.8 years in the above-mentioned exploratory trial, and 58.1 ± 15.5 years in the RECOVERY study. Studies have shown that older age is a risk factor for fatality related to COVID-19 (3, 27). Second, our study had a large proportion of patients with comorbidities, especially heart failure (39.6–47.9%) and renal insufficiency (39.6–41.7%). In the RECOVERY study, only about 18–19% of patients had heart disease and about 2% had severe kidney impairment (15). The discrepancy indicated that the patients in our study were in poorer health state, which may contribute to a worse prognosis and high mortality. Third, delayed use of antiviral drugs may also contribute to high morbidity. Most of our patients received antiviral drugs after intubation instead of in early phase of COVID-19, so maybe it was too late. It was well-known that early use of antiviral drugs lead to better prognosis (4, 5).

Except for the standard of care therapies, the additional treatments such as antiviral drugs, tocilizumab, or ECMO were left at the discretion of the physicians in the study. During the surge there was a national drug shortage, which made the use of additional treatments variable among patients. Our study found that patients in the baricitinib group were more likely to receive nirmatrelvir/ritonavir and IVIG. Nirmatrelvir/ritonavir could reduce risk of progression to severe COVID-19 and mortality for non-hospitalized adults, and its effectiveness on severe COVID-19 was unknown (5, 28). In the current study, most of patients received nirmatrelvir/ritonavir after intubated and admitted to ICU instead of within five days of the onset of symptoms, which was not supported by evidence. The effectiveness of delayed use of nirmatrelvir/ritonavir complicated matters even further. Regarding IVIG, available data do not support its use in severe COVID-19. In a multicenter, double-blind, placebo-controlled, phase 3 trial of patients with moderate-to-severe COVID-19-associated acute respiratory distress syndrome, IVIG did not improve clinical outcomes at day 28 and tended to be associated with an increased frequency of serious adverse events (29). Two retrospective studies reported similar results and found IVIG was not associated with significant changes in mortality in severe COVID-19 patients (30, 31). In conclusion, although published studies showed that neither nirmatrelvir/ritonavir nor IVIG reduce mortality in severe COVID-19, their effect on outcome were difficult to account for in a retrospective study design.

Due to PSM, most comorbidities were balanced between groups except hypertension. Our study reported that baricitinib group had a higher proportion of patients with hypertension. Hypertension has been identified as the most prevalent cardiovascular comorbidity in patients infected with COVID-19 (21). So far, the evidence had been mixed about the association between hypertension and COVID-19 prognosis (32–34). In a study conducted in Italy, hypertension was not an independent predictor of mortality in the multivariate analysis (24). Another European registry study, which included more than 9,000 patients, reported that hypertension was not independently related with in-hospital mortality (35). On the other hand, a large-scale analysis in China showed that hypertension was a significant risk factor for poor outcomes including admission to an intensive care unit, invasive ventilation, or death (21). A meta-analysis found that hypertension acted as an independent risk factor for deterioration of COVID-19 (36). So inconsistent results about association between hypertension and COVID-19 prognosis make the interpretation of our findings troublesome. The retrospective design of our study further made it difficult to clarify the impact of hypertension on the outcome.

The current study also found there were disagreement about CK and CK-MB in patients. Compared to baricitinib group, the control group had higher level of CK-MB and similar levels of CK. Previous results showed that the cardiovascular system of the COVID-19 patients had been notably damaged, and the degree of damage could be evaluated by cardiac biomarkers such as CK and CK-MB. Most studies showed that the elevated levels of CK and CK- MB were significantly associated with an increased risk of the mortality in COVID-19 infected patients (37). For example, in a retrospective analysis including 2954 COVID-19 patients, those with higher CK-MB had a significantly higher mortality rate compared to patients with normal levels (38). Two meta-analysis revealed that higher CK and CK-MB were associated with the mortality and severe disease in COVID-19 patients (39, 40). But there were some studies reporting conflicting results as well. A study found CK-MB and CK had no significant difference in the prediction effect of the mortality in COVID-19 (41). The disagreement of CK and CK-MB found in our study made it difficult to clarify whether patients in the control group had more severe cardiac damage than patients in the baricitinib group. As a result, the impact of cardiac damage on the outcome could be evaluated neither.

Some limitations of the study merit consideration. First, the present study was a retrospective study, so there were inherent problems related to this design. Second, we did not have data about the specific variant or sub-variant of each patient in our cohort. Third, some disparities in baseline characteristics and concomitant treatment were present which may have influenced the outcome parameters.

## Conclusion

The present study revealed baricitinib reduced 28-days mortality in severe COVID-19 patients on IMV. The effectiveness of baricitinib in treating patients with severe COVID-19 on IMV needs to be further investigated through future studies.

## Data Availability

The raw data supporting the conclusions of this article will be made available by the authors, without undue reservation.
